# Will higher traffic flow lead to more traffic conflicts? A crash surrogate metric based analysis

**DOI:** 10.1371/journal.pone.0182458

**Published:** 2017-08-07

**Authors:** Yan Kuang, Xiaobo Qu, Yadan Yan

**Affiliations:** 1 Griffith School of Engineering, Griffith University, Gold Coast, Queensland, Australia; 2 School of Civil and Environmental Engineering, University of Technology Sydney, Sydney, New South Wales, Australia; 3 School of Civil Engineering, Zhengzhou University, Zhengzhou, Henan, China; Beihang University, CHINA

## Abstract

In this paper, we aim to examine the relationship between traffic flow and potential conflict risks by using crash surrogate metrics. It has been widely recognized that one traffic flow corresponds to two distinct traffic states with different speeds and densities. In view of this, instead of simply aggregating traffic conditions with the same traffic volume, we represent potential conflict risks at a traffic flow fundamental diagram. Two crash surrogate metrics, namely, Aggregated Crash Index and Time to Collision, are used in this study to represent the potential conflict risks with respect to different traffic conditions. Furthermore, Beijing North Ring III and Next Generation SIMulation Interstate 80 datasets are utilized to carry out case studies. By using the proposed procedure, both datasets generate similar trends, which demonstrate the applicability of the proposed methodology and the transferability of our conclusions.

## Introduction

Due to the massive losses caused by road crashes, traffic safety has become a high-priority issue to traffic researchers and engineers [1–4]. As highlighted in an overview paper by Lord and Mannering [5], many generalized linear regression models were developed to establish the relationship between crash count, traffic parameters and road geometry parameters. Among such research, a considerable amount of pioneering studies have been conducted addressing the relationship between historical crash data and traffic volume, using different forms of traffic volume, such as Annual Average Daily Traffic (AADT), the hourly traffic volume and the volume/capacity (V/C) ratio [6–18]. Many different models have been developed to represent the relationship between the traffic volume and crash data, either linearly or non-linearly. Tanner [19] found that the traffic volume and crash data followed the model *Y* = *αF*^*β*^, where *Y* represented the crash count, *F* denoted the traffic volume, and *α*, *β* were the calibration coefficients. A non-linear concave function was found by Hauer and Persaud [20] to represent this relationship. Abbas [21] concluded that it followed a power function for most rural roads in Egypt. A typical U-shaped relationship was constructed in some other studies [22–25].

One particular traffic volume corresponds to two distinct traffic states with different densities and speeds in the fundamental diagram [26–28]. Although these two states have the same volume, it is of significance to distinguish them when analysing the relationship between traffic volume and crash. It is widely accepted that traffic volume itself is inappropriate to represent a traffic state [29–31]. Other traffic flow characteristics such as speed and/or density should be considered when establishing the relationship between traffic conditions and potential conflict risks. This relationship should be formulated based on the actual crash data. However, large amounts of quality crash data in some traffic conditions are usually absent in practice, making it difficult to represent flow—crash relationship cross all traffic conditions. Hence, in this research, we decide to use crash surrogate metrics to represent potential conflict risks, since crash surrogates have more frequent occurrence [32–37]. Many crash surrogate metrics have been proposed and applied to measure traffic conflict risks in existing studies [38–47].

We examine the relationship between traffic flow and potential conflict risk by using two prominent crash surrogate metrics, namely, Aggregated Crash Index and Time to Collision. Case studies are then carried out based on the Beijing North Ring III and Next Generation SIMulation Interstate 80 datasets. Using the proposed procedure, both datasets generate similar flow—conflict risk trends, which demonstrate the applicability of the proposed methodology and the transferability of our conclusions.

## Surrogate metrics

### Aggregated Crash Index (ACI)

The ACI is a tree-structured crash surrogate metric proposed by Kuang et al. [48], through imposing a hypothetical disturbance to the leading vehicle in a car-following scenario. Eight possible conflict types are defined by four levels of conditions, as shown in Table 1, where *d*_1_ is the degree of disturbance; *D*_1−2_(0) is the initial distance gap; *V*_1_(0) and *V*_2_(0) are the initial speed for leading and following vehicle respectively; Δ*V*(0) is the initial speed difference. The ACI represents the accumulation of the crash risk of all possible crash outcomes. Mathematically, the ACI can be represented as

$$ACI=\sum_{j=1}^{8}CR_{L_{j}}=\sum_{j=1}^{8}P\left(L_{j}\right)\cdot C_{L_{j}}$$ (1)

where $C_{L_{j}}$ and $CR_{L_{j}}$ are the crash risk and the crash outcomes incurred at each leaf node *L*_*j*_ of tree structure which is shown in Table 1. The ACI thus directly represents the potential conflict risk, considering additional factors such as reaction time and braking capacity. For any car-following scenario *i*, the ACI over the time period *T* can be represented as

$$ACI_{i}=\frac{\sum_{t=0}^{N}ACI_{i}\left(t\right)\cdot \Delta t}{T}$$ (2)

where *ACI*_*i*_(*t*) denotes the ACI value for the *i*th car-following scenario at discrete time *t*; *N* and Δ*t* are the total number and duration of the time intervals; *T* is the total time duration investigated, *T* = *N* ⋅ Δ*t*.

**Table 1:** Leaf nodes of the tree structure [48].

Conflict type	Condition level 1 *τ*^1^ = (*T*_1_ *vs*. *R*)	Condition level 2 *τ*^2^ = (*T*_*A*/*B*_ *vs*. *R*)	Condition level 3 *τ*^3^ = (*TTC*(*R*) *vs*. (*T*_1_ − *R*)/2)	Condition level 4 *τ*^4^ = (*BRAD*_1/2_ *vs*. *MADR*)	Leaf node *L*_*j*_	Probability *P*(*L*_*j*_)	Outcome **$C_{L_{j}}$**
**A1**	*R* ≥ *T*_1_	*R* ≥ *T*_*A*_	—	—	L_1_	P(L_1_)	1
**B1**	*R* < *T*_1_	*R* ≥ *T*_*B*_	—	—	L_4_	P(L_4_)	1
**A211**	*R* ≥ *T*_1_	*R* < *T*_*A*_	*TTC*(*R*) ≥ (*T*_1_ − *R*)/2	*BRAD*_1_ *> MADR*	L_2_	P(L_2_)	1
**A210**	*R* ≥ *T*_1_	*R* < *T*_*A*_	*TTC*(*R*) ≥ (*T*_1_ − *R*)/2	*BRAD*_1_ ≤ *MADR*	L_3_	P(L_3_)	0
**B211**	*R* < *T*_1_	*R* < *T*_*B*_	*TTC*(*R*) ≥ (*T*_1_ − *R*)/2	*BRAD*_1_ *> MADR*	L_6_	P(L_6_)	1
**B210**	*R* < *T*_1_	*R* < *T*_*B*_	*TTC*(*R*) ≥ (*T*_1_ − *R*)/2	*BRAD*_1_ ≤ *MADR*	L_5_	P(L_5_)	0
**B220**	*R* < *T*_1_	*R* < *T*_*B*_	*TTC*(*R*) < (*T*_1_ − *R*)/2	*BRAD*_2_ ≤ *MADR*	L_7_	P(L_7_)	0
**B221**	*R* < *T*_1_	*R* < *T*_*B*_	*TTC*(*R*) < (*T*_1_ − *R*)/2	*BRAD*_2_ *> MADR*	L_8_	P(L_8_)	1

Notations: *R*: reaction time of the following vehicle; *T*_1_: stopping time of leading vehicle; *T*_*A*_: value of $\frac{D_{1-2}(0)+\frac{V_{1}^{2}(0)}{2d_{1}}}{V_{2}(0)}$; *T*_*B*_: value of $\frac{\sqrt{\Delta V^{2}(0)+2d_{1}\cdot D_{1-2}(0)}-\Delta V(0)}{d_{1}}$; P(*L*_*j*_): probability of conflict *L*_*j*_ in car-following scenario *i*; *BRAD*: the minimum deceleration rate required to avoid a collision; *MADR*: the maximum available deceleration rate.

*BRAD*_1_ is suitable for the situation where the leading vehicle stops earlier than or at the same as the following vehicle, while *BRAD*_2_ works for the scenario where the following vehicle stops before the leading vehicle.

### Time to Collision (TTC)

Time to Collision (TTC) is another widely used crash surrogate metric, which is defined as the time remains until a collision between two vehicles would have occurred if the collision course and speed difference are maintained [38], mathematically,

$$TTC=\{\begin{array}{ll} \frac{D_{1-2}}{v_{2}-v_{1}}, & \text{if }v_{2}>v_{1}\\ \infty , & \text{otherwise} \end{array}$$ (3)

At a particular time, *D*_1−2_ represents the distance gap of the leading and following vehicles; *v*_1_ and *v*_2_ denote the speeds of the leading and following vehicles. This metric has been widely applied in evaluating the level of safety in different traffic situations [49, 50].

## Methodology

In this research, we intend to demonstrate potential conflict risks with respect to different traffic conditions in a traffic flow fundamental diagram. In this regard, we need to divide all data into many distinct traffic conditions with different flow characteristics and conflict risks. Firstly, the traffic conflict risk of each car-following scenario can be estimated by using the ACI and TTC, based on the collected traffic data (i.e., speeds, vehicle lengths and time headways). Those original traffic data of car-following scenarios are then integrated into many aggregated points with respect to different traffic conditions. The potential conflict risk of each aggregated traffic point is calculated based on the number of car-following scenarios involved in each 60-second time period. Finally, those aggregated traffic points are divided into many traffic states, sorted by density, with uniform span.

### Traffic conflict data

As suggested by previous researchers [51, 52], the distance headway for a car-following scenario *D*_1−2_ can be estimated by (*V*_2_ × *h*_2_ − *l*_1_). *l*_1_ denotes the length of the leading vehicle in the car-following scenario, while *V*_2_ and *h*_2_ represent the speed and time headway of the following vehicles at a particular time, respectively.

As our road section is rather short, we assume the traffic flow in this road section is homogeneous with a similar traffic flow characteristics. In other words, we can simply measure the potential conflict risk at a particular spot, and use aggregated risk over time to represent the risk values for this short road section in the given time period. In this research, ACI can represent the risk directly, while the risks represented by TTC are determined by comparing their values and thresholds. Mathematically,

$$IR_{ij}=\{\begin{array}{ll} S_{j}{}^{*}-S_{ij}\text{,} & \text{if }S_{j}{}^{*}>S_{ij}\\ 0, & \text{otherwise} \end{array}$$ (4)

where *IR*_*ij*_ represents the Individual Risk (IR) of discrete car-following scenario *i* measured by surrogate *j*; *S*_*ij*_ denotes the surrogate value for discrete scenario *i* measured by surrogate *j*; $S_{j}^{*}$ is the threshold of surrogate metric *j*. As suggested by Kuang et al. [46, 48], the thresholds of TTC and ACI are adopted as 4s and 0, respectively. Accordingly, the traffic conflict risk of each car-following scenario can be measured by using surrogate metrics ACI and TTC.

### Aggregated traffic points

Each aggregated traffic point is calculated based on the number of vehicles passing through the road section in each 60-second time period. The aggregated traffic density, speed and conflict refer to the average values of the source data involved in each time period. Similarly, the integrated conflict risk of each aggregated traffic point can be represented by the ACI and TTC, respectively.

### Traffic states

In order to examine the relationship between traffic conflict risk and traffic state, we adopt the following procedure to divide the field data into many traffic states, sorted by density, with uniform span.

Step 1 Rank all observations according to their densities, from smallest to largest,
  $$\left(k_{\left(1\right)},v_{\left(1\right)},f_{\left(1\right)},IR_{\left(1\right)}\right),\;\cdots ,\left(k_{\left(i\right)},v_{\left(i\right)},f_{\left(i\right)},IR_{\left(i\right)}\right),\cdots ,\;\left(k_{\left(m\right)},v_{\left(m\right)},f_{\left(m\right)},IR_{\left(m\right)}\right)$$ (5)
  where *k*_(1)_ ≤ ⋯ ≤ *k*_(*i*)_ ≤ ⋯ ≤*k*_(*m*)_, and *v*_(*i*)_, *f*_(*i*)_ and *IR*_(*i*)_ are, respectively, the corresponding speed, flow rate and IR value under traffic density *k*_(*i*)_.
Step 2 Determine the total number of intervals for those observations with constant span *δ* which is set as 1.5 veh/km, mathematically
  $$\tilde{n}=\text{round}\left(\frac{k_{\text{max}}-k_{\text{min}}}{\delta }\right)$$ (6)
  where $\tilde{n}$ is the total number of intervals, and *k*_max_ and *k*_min_ represent the maximum and minimum density of the data, respectively.
Step 3 Find the range of each interval as follows:
  $$(k_{\text{min}}+\delta \cdot \left(n-1\right),\;k_{\text{min}}+\delta \cdot n],\;n\in \left(1:1:\tilde{n}\right)$$ (7)
  Then count and record the number of data points for the corresponding interval as
  $$N_{n},\;n\in \left(1:1:\tilde{n}\right)$$ (8)
Step 4 Calculate the Cumulative Risk (CR) value for each interval, which can be written as
  $$CR_{n}=\sum_{i=M_{n-1}}^{M_{n-1}+N_{n}}IR_{i},\;M_{n-1}=\sum_{n=1}^{n-1}N_{n},\;n\in \left(1:1:\tilde{n}\right)$$ (9)
  where *CR*_*n*_ denotes the CR value for the *n*th interval, while *M*_*n*−1_ and *IR*_*i*_ respectively denote the lower bound of the *n*th interval and the *i*th IR value in step 1.
Step 5 Compute the Average Risk (AR) value for each interval as follows:
  ARn=CRnNn,  n∈(1:1:n˜) (10)
  where *AR*_*n*_ and *N*_*n*_ are the AR and the total number of *i* for the *n*th interval.

## Data collection

Two distinct datasets are used to demonstrate our potential risk flow relationship. Data I was collected on the North Ring III expressway in Beijing, China, and data II was gathered from the interstate 80 freeway in the San Francisco Bay area in Emeryville, CA, USA.

### Data I—Beijing North Ring III expressway

Data I (S1 File) was collected on June 21st (Tuesday) in 2011, during eight different time periods including two morning peak hours, three afternoon peak hours, and three off-peak hours. There are three lanes with speed limit of 80 km/hour at the spot. During the data collection, there were no traffic crash occurred. By taking advantage of the video recording systems, the traffic volumes, density spot speeds, time headways and vehicle lengths were recorded. Total 52,994 original car-following scenarios are used. Those original data are moreover integrated into 2,280 aggregated traffic data samples.

### Data II—NGSIM interstate 80 freeway

The data of Interstate 80 (I-80) Freeways was collected by the Next Generation SIMulation (NGSIM) program on eastbound I-80 in the San Francisco Bay area in Emeryville, CA, USA on April 13, 2005. As stated by [53], the study area was approximately 500 meters (1,640 feet) in length and consisted of six freeway lanes, including a high-occupancy vehicle (HOV) lane, with speed limit of 110 km/h. A total of 45 minutes of trajectory data are available in the full dataset, segmented into three 15-minute periods: 4:00 p.m. to 4:15 p.m.; 5:00 p.m. to 5:15 p.m.; and 5:15 p.m. to 5:30 p.m., by using seven cameras. We further extracted total 139,230 car-following scenarios from the trajectory data. 4,644 aggregated traffic data samples are generated on the basis of those source car-following scenarios data.

**Fig 1:**
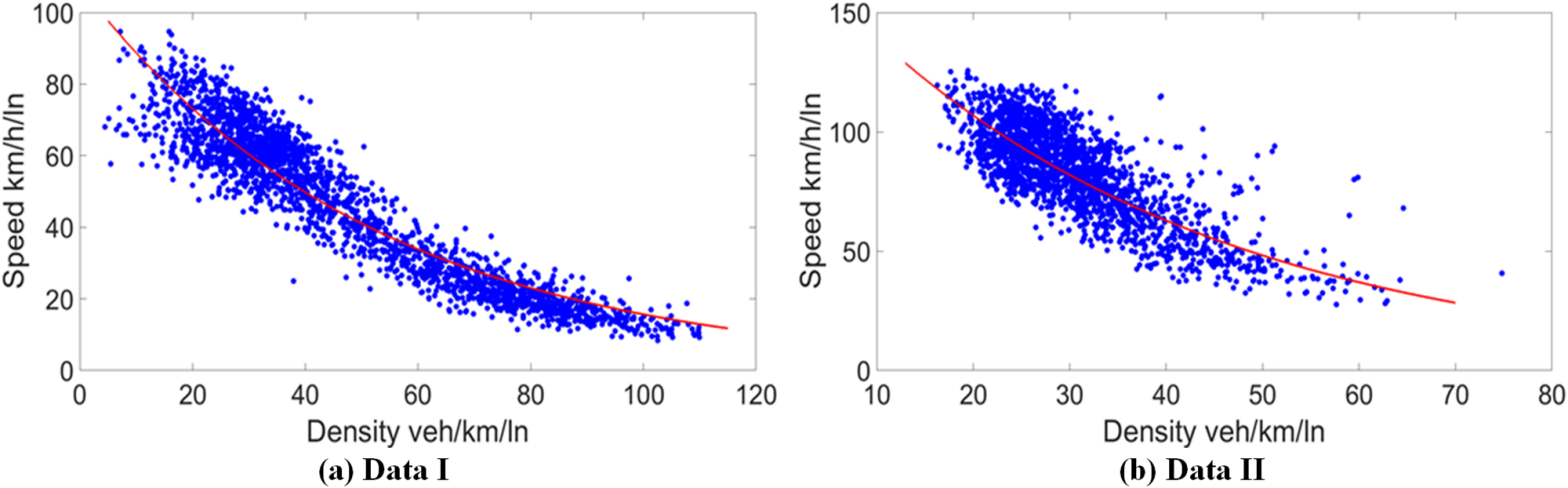
Aggregated traffic data in speed-density diagram for data I and II.

It is found that the speed and density of both data follow the Underwood model [54]. The free flow speed and capacity density are estimated as 107.6 km/h and 51.9 veh/km, 181.8 km/h and 37.7 veh/km for data I and II, respectively. Fig 1 shows the characteristics of the aggregated data for both datasets in a speed-flow fundamental diagram. As can be seen in Fig 1, two data have distinct characteristics and distributions in speed and density. Data I is evenly distributed from density 20 veh/km to 100 veh/km, while data II is more concentrated in the range of density 20 veh/km to 40 veh/km. Those distinct flow characteristics are caused by the different speed limit, data collection time period and traffic conditions.

## Potential risk—Flow relationship

By using the methodology mentioned, the traffic states can be generated by using the aggregated traffic points. Accordingly, the density, flow, speed and conflict risk of each traffic state is estimated based on those characters of aggregated traffic points. Regarding each interval as a unique traffic state, total 70 and 39 traffic states are generated for data I and II, respectively. All traffic states are distinguished by their traffic flow characteristics and AR represented by the ACI and TTC. In order to better demonstrate the relationship between conflicts and traffic states, the curve of speed and flow are generated based on the Underwood model obtained previously. We further map the risk of each state to the corresponding position on the speed-density curve, using the AR value to determine the size and color of each point. Accordingly, the traffic conflict risk can be visually mapped onto the corresponding position in the speed-density fundamental diagram.

**Fig 2:**
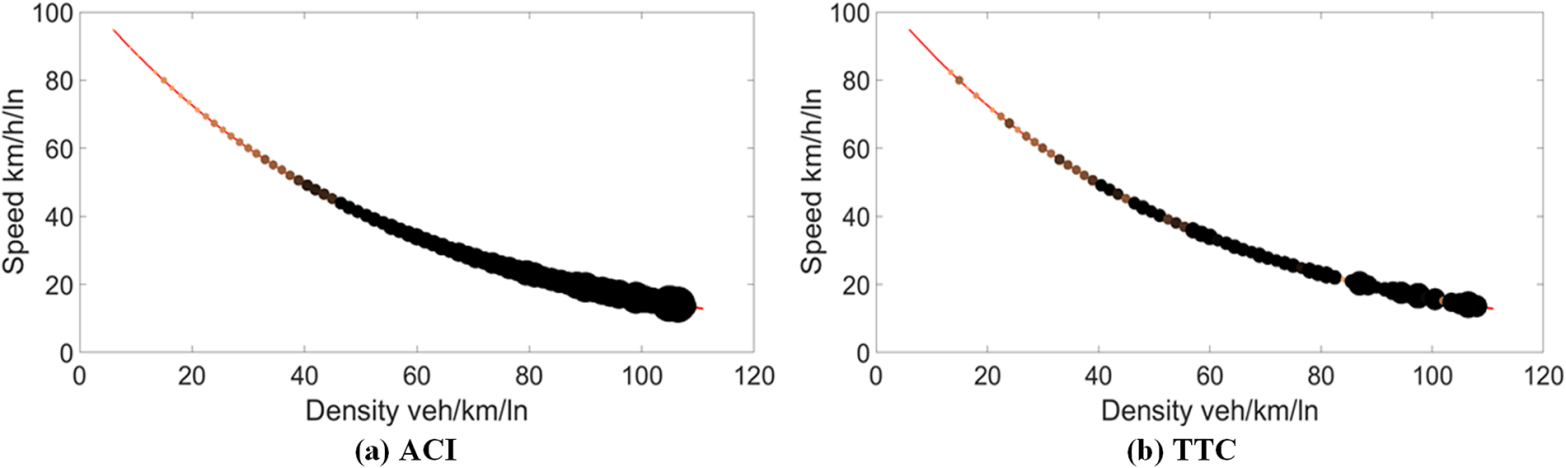
Traffic conflict risk on speed-density curve for data I.

**Fig 3:**
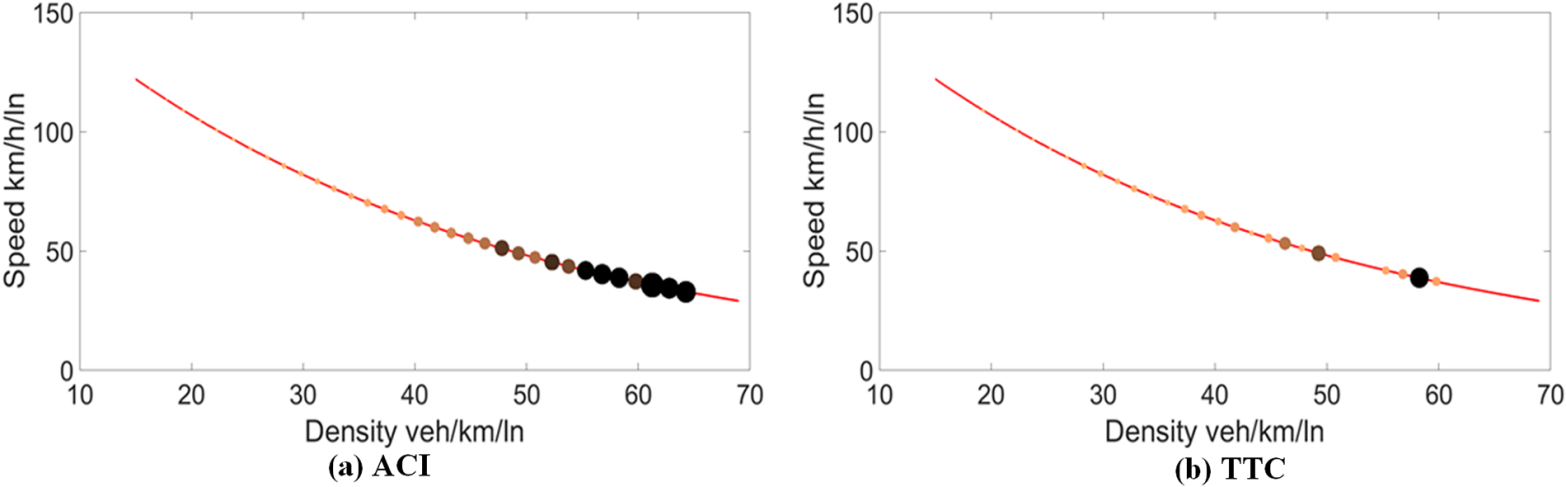
Traffic conflict risk on speed-density curve for data II.

Fig 2 shows the speed-density diagram mapped with traffic conflict risk represented by the ACI and TTC, respectively. The bigger size and darker color of a point indicate the higher potential conflict risk of a traffic state. Obviously, the traffic conflict risk increases with an increase in density and a decrease in speed. When the density increases, the distance headways between cars are reduced, with a potential to increase the risk even though the speed decreases. As can be seen in Figs 2 and 3, the ACI has more obvious trend on the change of risks than the TTC for both data.

**Fig 4:**
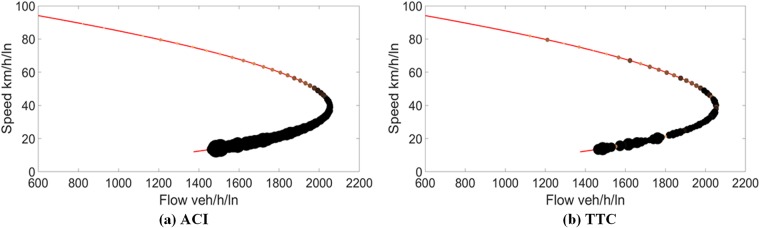
Traffic conflict risk on the speed-flow curve for data I.

**Fig 5:**
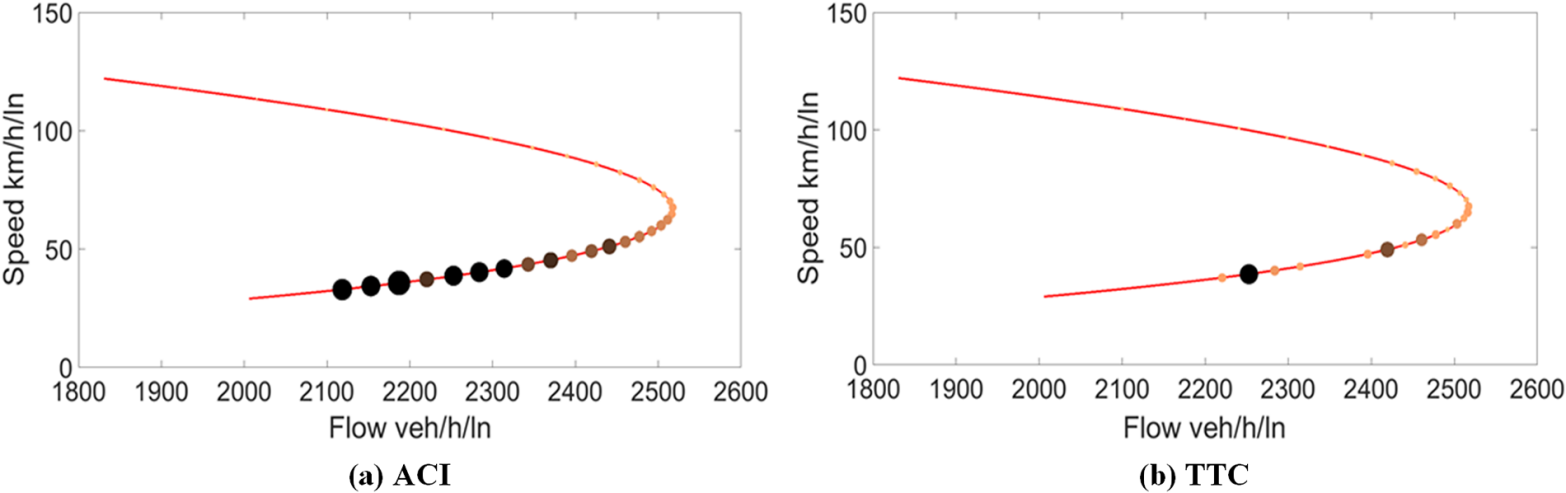
Traffic conflict risk on the speed-flow curve for data II.

We further use the same method to map the traffic conflict risk of different states on the speed-density curve, using the risk represented by the ACI and TTC to determine the size and colour of each point. Figs 4 and 5 show the fundamental diagrams mapped with traffic conflict risk represented by the ACI and TTC, for data I and II, respectively.

As depicted in Figs 4 and 5, the points are found with size increased and colour thickened as we move from top to bottom. Obviously, the potential conflict risks measured by the ACI and TTC increase when the speed decreases. As can be seen in Fig 4, the flow value located between 1,500 veh/h and capacity is found corresponding to two traffic states, with different speeds. Apparently, the traffic conflict risk is found to be distinct for those two states, the traffic conflict risk of the capacity state clearly being less than that of the over-capacity state. As can be seen in Fig 5, the conflict risk is increasing when the speed decreases along the curve from the top to the bottom in both graphs. There are two distinct traffic states found between flow rate 2,200 veh/h and capacity. The trend is also found for data II in Fig 5 by using the ACI and TTC. Thus, we can conclude that the traffic conflict risk increases along the curve, from level of service A to F. Besides, it is found that the ACI can represent the change of risks better than TTC for both data. This is consistent with that ACI has better performance than surrogate TTC by Kuang et al. [48]. The possible reasons are as follows: (1) The ACI can evaluate more car-following scenarios than TTC. According to the notions of TTC, all scenarios in which the speed of the following vehicle is not greater than that of the leading vehicle are regarded as safe. That is to say, the condition used to determine the risk is the speed differential. Thus, it is impossible to identify the risks in other scenarios by using the TTC. The ACI is based on a hypothetical disturbance; it can be used in any car-following scenario, even the leading vehicle’s speed is greater than that of the following vehicle. Therefore, the ACI can have more accurate results than TTC. (2) The ACI takes more important variables into account. Since the drivers and vehicles are most critical parts in crash mechanism, the consideration of the driver’s reaction time and the MADR can contribute to a better representation of the risk.

## Discussion and conclusions

Since one traffic volume corresponds to two different traffic states with different speeds and densities, traffic flow itself is not able to properly represent a traffic condition. In this regard, we visualize a potential traffic risk—flow relationship in a traffic flow fundamental diagram. To this end, we use two classical surrogate metrics, namely, ACI and TTC, to represent the potential conflict risk in this study. Two distinct datasets, one collected in China and the other gathered in the U.S., are used to testify our methodology. Based on the case studies, the potential conflict risk is found to have a strong correlation with the level of service, as the latter increases from level A to F. The curves for both case studies have a similar pattern, which demonstrate the transferability of the proposed methodology.

This research has two limitations. Firstly, the sample size and data quality might not be large enough to conclude the above findings. In the future work, more data will be collected to validate the relationship that is presented in the potential risk flow relationship. Moreover, micro-simulation could be used to generate simulated data for further analysis. Secondly, the study is based on the assumption that the potential conflict risk has a strong correlation with crash frequency. As a follow up study, this relationship will be validated properly using actual crash data.

## Supporting information

S1 File Data I collected from Beijing expressway. (XLSX)
